# Prediction model and significance of myocardial injury induced by fluorouracil combined with platinum-based chemotherapy in advanced gastric cancer based on baseline data and inflammation-nutrition-atherosclerosis factors

**DOI:** 10.3389/fmed.2025.1700554

**Published:** 2025-11-18

**Authors:** Tian Zhang, Feifei Kong, Lei Cao, Lanhua Zhao

**Affiliations:** 1 Department of Medical Oncology, The Affiliated Hospital of Xuzhou Medical University, Xuzhou, China; 2 Department of Integrated Traditional Chinese and Western Medicine Oncology, The Affiliated Hospital of Xuzhou Medical University, Xuzhou, China

**Keywords:** advanced gastric cancer, fluorouracil, platinum-based drugs, myocardial injury, predictive model

## Abstract

**Objective:**

To develop and evaluate a predictive model for myocardial injury in patients with advanced gastric cancer treated with fluorouracil plus platinum-based chemotherapy, incorporating baseline characteristics and inflammatory, nutritional, and atherosclerotic factors.

**Methods:**

A total of 268 patients with advanced gastric cancer who received this treatment between April 2020 and September 2024 were selected and divided into a training set (*n* = 188) and a validation set (*n* = 80) in a 7:3 ratio. In the training set, multivariate logistic regression analysis was used to identify risk factors for myocardial injury in patients with advanced gastric cancer treated with fluorouracil and platinum-based drugs, and a nomogram prediction model was constructed. The predictive model’s performance was evaluated using receiver operating characteristics (ROC) curves and calibration curves, and the model was validated in the validation set. Additionally, decision curve analysis (DCA) was performed to assess clinical utility.

**Results:**

In the training set, 56 patients (29.79%) developed myocardial injury, while 23 patients (28.75%) in the validation set developed myocardial injury, with no statistically significant difference in the incidence or clinical characteristics between the two sets (*p* > 0.05). In the training set, age, hypertension, serum C-reactive protein (CRP), interleukin-6 (IL-6), tumor necrosis factor-α (TNF-α), serum albumin, prealbumin, lipoprotein(a) (Lp(a)), and homocysteine (Hcy) were identified as influencing risk factors (all *p* < 0.05), and a nomogram prediction model was constructed. The model demonstrated good calibration and fit in both the training and validation sets (*C*-index: 0.901 and 0.9879, respectively; mean absolute errors between predicted and actual values: 0.133 and 0.115, respectively; Hosmer–Lemeshow test *p*-values: 0.136 and 0.669, respectively). ROC curve analysis showed that the AUCs for predicting myocardial injury in the training and validation sets were 0.901 (95% CI: 0.823–0.978) and 0.879 (95% CI: 0.819–0.938), respectively, with sensitivities and specificities of 0.756, 1.000 and 0.703, 0.951, respectively.

**Conclusion:**

This predictive model aids in the early identification of myocardial injury, guiding clinical decision-making and improving prognosis.

## Introduction

Gastric cancer was one of the most common malignant tumors worldwide, posing a serious threat to human health (1). The combination of fluorouracil and platinum-based drugs was a commonly used chemotherapy regimen for advanced gastric cancer, which can prolong patient survival to some extent. However, this regimen carries cardiotoxicity and may lead to myocardial injury, adversely affecting patients’ quality of life and prognosis (2). Early identification of high-risk factors for myocardial injury and the development of effective predictive models were of great significance in preventing and reducing the occurrence of myocardial injury, as well as optimizing treatment strategies (3). Currently, although some studies have focused on chemotherapy-related myocardial injury, the construction and evaluation of predictive models for myocardial injury in patients with advanced gastric cancer treated with fluorouracil combined with platinum-based drugs remain inadequate. This study aimed to analyze baseline data and inflammation-nutrition-atherosclerosis factors to develop a predictive model and assess its clinical value, thereby providing a reference for clinical practice.

## Materials and methods

### Study subjects

A total of 268 patients with advanced gastric cancer who received fluorouracil combined with platinum-based chemotherapy in the hospital from April 2020 to September 2024 were selected. Inclusion criteria: Pathologically confirmed diagnosis of advanced gastric cancer. Age 18–75 years. Treated with fluorouracil combined with platinum-based chemotherapy. Patients with complete data for all variables of interest. Signed informed consent. Exclusion criteria: Comorbid severe cardiac diseases (e.g., acute myocardial infarction, severe arrhythmia, etc.). Severe liver or kidney dysfunction; Mental illness rendering the patient unable to cooperate with the study. Recent use of medications affecting cardiac function.

### Grouping method

A complete randomization method was employed, using a random number table to divide the patients into a training set (*n* = 188) and a validation set (*n* = 80) in a 7:3 ratio. The training set was used to screen risk factors and construct the prediction model, while the validation set was used to verify the predictive performance of the model.

### Data collection

Baseline data of the patients were collected, including age, gender, body mass index (BMI), smoking history, alcohol consumption history, comorbid hypertension, comorbid diabetes, history of coronary heart disease, and chronic renal insufficiency. Laboratory indicators before chemotherapy were measured, such as complete blood count, liver and kidney function, blood lipids, blood glucose, serum C-reactive protein (CRP), interleukin-6 (IL-6), tumor necrosis factor-α (TNF-α), serum albumin, prealbumin, lipoprotein a (Lp(a)), homocysteine (Hcy), creatine kinase-MB (CK-MB), N-terminal pro-B-type natriuretic peptide (NT-proBNP), high-sensitivity CRP (hs-CRP), D-dimer, fibrinogen, erythrocyte sedimentation rate (ESR), hemoglobin, platelet count, lymphocyte count, neutrophil count, and neutrophil-to-lymphocyte ratio (NLR). Cardiac function indicators, such as electrocardiograms (ECG) and cardiac ultrasound, were regularly monitored during chemotherapy, and the occurrence of myocardial injury was determined based on relevant criteria. New ischemic ST-T changes or arrhythmias on ECG, confirmed by blinded assessment from at least two cardiologists, could serve as supporting diagnostic evidence but were insufficient for diagnosis alone.

In this study, both intravenous and oral formulations of fluorouracil were used, with the choice of administration based on the patient’s specific clinical condition. For oral fluorouracil (capecitabine), the standard dose was 1,000–1,250 mg/m^2^/day; for patients with creatinine clearance <60 mL/min or serum albumin <35 g/L, the dose was adjusted to 750–1,000 mg/m^2^/day. Intravenous fluorouracil (5-FU) was administered via 24-h continuous infusion at a dose of 2,400–3,000 mg/m^2^ every 2 weeks. The specific dose and administration method were recorded for each patient. For platinum-based drugs, oxaliplatin was selected due to its widespread use in chemotherapy for advanced gastric cancer. Note: Due to initial study design limitations, detailed information on oxaliplatin dose delays/reductions caused by non-cardiac toxicities was not systematically recorded.

To determine the occurrence of myocardial injury, we primarily relied on regular monitoring of cardiac function indicators during chemotherapy, including ECG, cardiac ultrasound, and laboratory markers such as cTn, CK-MB, and NT-proBNP. Referring to the Fourth Universal Definition of Myocardial Infarction (2018) (4), myocardial injury was primarily diagnosed when blood cTn levels exceeded the 99th percentile upper reference limit (URL) (cTnI 99th percentile URL: 0.04 ng/mL). ECG abnormalities (e.g., ST-segment changes, arrhythmias), cardiac ultrasound impairment (e.g., decreased LVEF, structural abnormalities), and elevated CK-MB (>25 U/L)/NT-proBNP (>125 pg/mL for age <75 years, >450 pg/mL for age ≥75 years) served as auxiliary diagnostic criteria to confirm myocardial injury. This standardized and comprehensive assessment ensured accurate identification of chemotherapy-induced myocardial injury. Monitoring of cardiac function indicators was performed before chemotherapy and after each chemotherapy cycle (once every 3 weeks). Myocardial injury was diagnosed at the first monitoring timepoint when the diagnostic criteria were met, and the specific chemotherapy cycle of diagnosis was recorded for each patient.

### Statistical analysis

Statistical analysis was performed using SPSS 25.0 and R 4.0.3. Measurement data were expressed as mean ± standard deviation ( $\bar{x}$ ± s), and intergroup comparisons were conducted using independent samples t-tests. Count data were expressed as numbers and percentages (*n*, %), and intergroup comparisons were conducted using *χ*^2^ tests. Univariate analysis and multivariate logistic regression analysis were used to screen risk factors and construct the prediction model, and their odds ratios (OR) and 95% confidence intervals (CI) were calculated. Variance inflation factors (VIF) were calculated to exclude multicollinearity (VIF threshold <10). The “rms” package in R was used to construct the nomogram model. The receiver operating characteristic (ROC) curve was plotted, and the area under the curve (AUC) value was calculated. Internal validation of the model was performed using the bootstrap method, and calibration curves comparing predicted and actual results were plotted. The concordance index (*C*-index) was calculated, and the Hosmer–Lemeshow test was used to evaluate the goodness-of-fit of the prediction model. Given the number of predictors, the potential for overfitting was assessed using bootstrap validation (1,000 resamples) to calculate an optimism-corrected concordance index. Decision curve analysis (DCA) was performed to assess the clinical application value of the model. A *p* < 0.05 was considered statistically significant.

## Results

### Comparison of general clinical characteristics between training and validation sets

A total of 268 patients with advanced gastric cancer were selected and divided into a training set (*n* = 188) and a validation set (*n* = 80). There were no statistically significant differences between the training and validation sets in baseline data such as age, gender, height, weight, smoking history, alcohol consumption history, comorbid hypertension, comorbid diabetes, history of coronary heart disease, chronic renal insufficiency, or chemotherapy regimens, indicating comparability (*p* > 0.05) (Table 1).

**Table 1:** Comparison of general data and clinical characteristics between the training and validation sets.

Indicators		Training set (*n* = 188)	Validation set (*n* = 80)	Statistical value	*p*
Age (years)		55.68 ± 8.23	54.97 ± 7.86	0.654	0.513
Gender	Male	102 (54.26)	42 (52.50)	0.069	0.791
	Female	86 (45.74)	38 (47.50)		
BMI (kg/m^2^)		23.56 ± 2.87	23.38 ± 2.73	0.476	0.634
Smoking history	Yes	62 (32.98)	23 (28.75)	0.463	0.496
	No	126 (67.02)	57 (71.25)		
Alcohol history	Yes	56 (29.79)	21 (26.25)	0.342	0.558
	No	132 (70.21)	59 (73.75)		
Hypertension	Yes	46 (24.47)	18 (22.50)	0.119	0.729
	No	142 (75.53)	62 (77.50)		
Diabetes	Yes	47 (25.00)	17 (21.25)	0.434	0.510
	No	141 (75.00)	63 (78.75)		
History of CAD	Yes	42 (22.34)	17 (21.25)	0.038	0.843
	No	146 (77.66)	63 (78.75)		
Chronic renal insufficiency	Yes	46 (24.47)	19 (23.75)	0.015	0.900
	No	142 (75.53)	61 (76.25)		
Hemoglobin (g/L)		125.63 ± 16.24	124.89 ± 14.98	0.349	0.727
Platelet count (×10^9^/L)		215.36 ± 35.67	213.48 ± 34.89	0.397	0.691
Lymphocyte count (×10^9^/L)		1.89 ± 0.56	1.82 ± 0.53	0.951	0.342
Neutrophil count (×10^9^/L)		4.23 ± 1.24	4.18 ± 1.16	0.307	0.758
NLR		2.34 ± 0.87	2.29 ± 0.83	0.436	0.662
ALT (U/L)		32.56 ± 10.23	31.87 ± 9.86	0.510	0.610
AST (U/L)		35.68 ± 11.34	34.91 ± 10.98	0.513	0.608
Creatinine (μmol/L)		78.65 ± 12.56	77.98 ± 12.14	0.403	0.686
Total cholesterol (mmol/L)		4.86 ± 0.87	4.82 ± 0.83	0.349	0.727
Triglycerides (mmol/L)		1.69 ± 0.54	1.62 ± 0.51	0.987	0.324
LDL-C (mmol/L)		2.87 ± 0.69	2.84 ± 0.62	0.335	0.737
HDL-C (mmol/L)		1.18 ± 0.23	1.13 ± 0.21	1.670	0.096
Blood glucose (mmol/L)		5.36 ± 1.02	5.32 ± 0.98	0.297	0.766
CRP (mg/L)		6.91 ± 2.56	6.78 ± 2.48	0.383	0.701
IL-6 (pg/mL)		19.06 ± 5.97	18.13 ± 5.18	1.212	0.226
TNF-α (pg/mL)		25.36 ± 7.89	23.98 ± 7.56	1.326	0.185
Serum albumin (g/L)		41.56 ± 3.24	40.78 ± 3.13	1.821	0.069
Prealbumin (mg/L)		256.34 ± 35.67	254.51 ± 34.98	0.386	0.699
Lp(a) (mg/dL)		29.96 ± 10.23	27.34 ± 9.86	1.939	0.053
Hcy (μmol/L)		12.56 ± 3.24	12.38 ± 3.12	0.420	0.674
CK-MB (U/L)		19.69 ± 5.67	18.22 ± 5.48	1.961	0.051
NT-proBNP (pg/mL)		125.63 ± 35.76	124.85 ± 34.89	0.164	0.864
hs-CRP (mg/L)		3.56 ± 1.24	3.52 ± 1.18	0.245	0.806
D-Dimer (mg/L)		0.37 ± 0.12	0.34 ± 0.11	1.918	0.056
Fibrinogen (g/L)		3.31 ± 0.56	3.22 ± 0.53	1.223	0.222
ESR (mm/h)		19.34 ± 5.67	18.03 ± 5.48	1.748	0.081
ECG abnormalities	Yes	26 (13.83)	9 (11.25)	0.328	0.566
	No	162 (86.17)	71 (88.75)		
Echocardiographic abnormalities	Yes	29 (15.43)	7 (8.75)	2.150	0.142
	No	159 (84.57)	73 (91.25)		

### Univariate analysis of myocardial injury in the training set

Univariate analysis in the training set showed that age, hypertension, CRP, IL-6, TNF-α, serum albumin, prealbumin, Lp(a), and Hcy were significantly associated with myocardial injury (*p* < 0.05), while other indicators showed no significant differences (all *p* > 0.05) (Table 2). Using myocardial injury occurrence as the dependent variable (no = 0, yes = 1) (Table 3). The results of multivariate logistic regression analysis indicated that age, hypertension, CRP, IL-6, TNF-α, serum albumin, prealbumin, Lp(a), and Hcy were significantly associated with myocardial injury in patients with advanced gastric cancer treated with fluorouracil and platinum-based chemotherapy (all *p* < 0.05). In the regression model, all variables had tolerance >0.1, VIF <10, and condition index <30, with no multicollinearity (no variance proportion >50% for multiple covariates under the same eigenvalue). Statistical tests confirmed no significant interaction effects between age and the other factors, indicating their independent contributions to myocardial injury risk. Additionally, no collinearity was found between CRP and IL-6 (Table 4).

**Table 2:** Univariate analysis of myocardial injury in the training set.

Indicator		Myocardial injury occurred (*n* = 56)	No myocardial injury occurred (*n* = 132)	Statistical value	*p*
Age (years)		58.21 ± 8.41	54.17 ± 7.98	3.123	0.002
Gender	Male	33 (58.93)	69 (52.27)	0.701	0.402
	Female	23 (41.07)	63 (47.73)		
BMI (kg/m^2^)		23.54 ± 2.91	23.69 ± 2.84	0.328	0.742
Smoking history	Yes	23 (41.07)	39 (29.55)	2.363	0.124
	No	33 (58.93)	93 (70.45)		
Alcohol history	Yes	18 (32.14)	38 (28.79)	0.211	0.645
	No	38 (67.86)	94 (71.21)		
Hypertension	Yes	22 (39.29)	24 (18.18)	9.475	0.002
	No	34 (60.71)	108 (81.82)		
Diabetes	Yes	16 (28.57)	31 (23.48)	0.542	0.461
	No	40 (71.43)	101 (76.52)		
History of CAD	Yes	14 (25.00)	28 (21.21)	0.325	0.568
	No	42 (75.00)	104 (78.79)		
Chronic renal insufficiency	Yes	17 (30.36)	29 (22.00)	1.496	0.221
	No	39 (69.64)	103 (78.00)		
Hemoglobin (g/L)		124.89 ± 15.87	125.91 ± 16.43	0.393	0.694
Platelet count (×10^9^/L)		214.56 ± 34.89	215.82 ± 36.01	0.221	0.825
Lymphocyte count (×10^9^/L)		1.82 ± 0.54	1.93 ± 0.57	1.228	0.220
Neutrophil count (×10^9^/L)		4.27 ± 1.21	4.19 ± 1.26	0.402	0.687
NLR		2.38 ± 0.84	2.32 ± 0.89	0.429	0.667
ALT (U/L)		32.23 ± 10.11	32.71 ± 10.46	0.290	0.771
AST (U/L)		35.32 ± 11.12	35.90 ± 11.56	0.4224	0.673
Creatinine (μmol/L)		78.34 ± 12.46	78.86 ± 12.73	0.257	0.796
Total cholesterol (mmol/L)		4.84 ± 0.78	4.87 ± 0.89	0.219	0.826
Triglycerides (mmol/L)		1.67 ± 0.52	1.74 ± 0.56	0.800	0.424
LDL-C (mmol/L)		2.85 ± 0.66	2.88 ± 0.71	0.270	0.787
HDL-C (mmol/L)		1.16 ± 0.22	1.19 ± 0.24	0.803	0.423
Blood glucose (mmol/L)		5.34 ± 1.01	5.37 ± 1.03	0.183	0.854
CRP (mg/L)		8.23 ± 2.67	6.32 ± 2.34	4.904	0.001
IL-6 (pg/mL)		22.34 ± 6.23	17.56 ± 5.67	5.131	0.001
TNF-α (pg/mL)		27.04 ± 8.21	23.67 ± 7.34	2.777	0.006
Serum albumin (g/L)		41.17 ± 3.01	42.34 ± 3.32	2.270	0.024
Prealbumin (mg/L)		245.67 ± 33.54	261.23 ± 36.78	2.721	0.007
Lp(a) (mg/dL)		33.14 ± 11.02	28.01 ± 9.56	3.212	0.001
Hcy (μmol/L)		13.54 ± 3.56	11.89 ± 3.41	2.994	0.003
CK-MB (U/L)		20.23 ± 5.89	19.34 ± 5.56	0.986	0.325
NT-proBNP (pg/mL)		130.32 ± 38.21	123.54 ± 33.67	1.212	0.227
hs-CRP (mg/L)		3.67 ± 1.32	3.49 ± 1.12	0.954	0.341
D-Dimer (mg/L)		0.39 ± 0.13	0.36 ± 0.11	1.617	0.107
Fibrinogen (g/L)		3.42 ± 0.59	3.29 ± 0.53	1.486	0.138
ESR (mm/h)		20.07 ± 6.01	18.56 ± 5.34	1.707	0.089
ECG abnormalities	Yes	8 (14.29)	18 (13.64)	0.013	0.906
	No	48 (85.71)	114 (86.36)		
Echocardiographic abnormalities	Yes	13 (23.21)	16 (12.12)	3.708	0.054
	No	43 (76.79)	116 (87.88)		

**Table 3:** Variable assignment method.

Variable	Meaning	Assignment
X1	Age	Continuous variable
X2	Comorbid hypertension	No = 0, Yes = 1
X3	CRP	Continuous variable
X4	IL-6	Continuous variable
X5	TNF-α	Continuous variable
X6	Serum albumin	Continuous variable
X7	Prealbumin	Continuous variable
X8	Lp(a)	Continuous variable
X9	Hcy	Continuous variable
Y	Occurrence of myocardial injury	No = 0, Yes = 1

**Table 4:** Multivariate logistic regression analysis of myocardial injury.

Indicator	*B*	Standard error	Wald	*p*	OR	95% CI
Age	0.063	0.027	5.298	0.021	1.065	1.009–1.124
Comorbid hypertension	1.179	0.463	6.494	0.011	3.250	1.313–8.047
CRP	0.255	0.092	7.589	0.006	1.290	1.076–1.546
IL-6	0.156	0.041	14.226	0.001	1.169	1.078–1.267
TNF-α	0.058	0.028	4.322	0.038	1.059	1.003–1.119
Serum albumin	−0.141	0.072	3.860	0.049	0.868	0.754–1.000
Prealbumin	−0.014	0.006	5.473	0.019	0.986	0.974–0.998
Lp(a)	0.062	0.022	8.373	0.004	1.064	1.020–1.111
Hcy	0.185	0.064	8.263	0.004	1.203	1.061–1.365

### Development of a nomogram prediction model for myocardial injury

**Figure 1:**
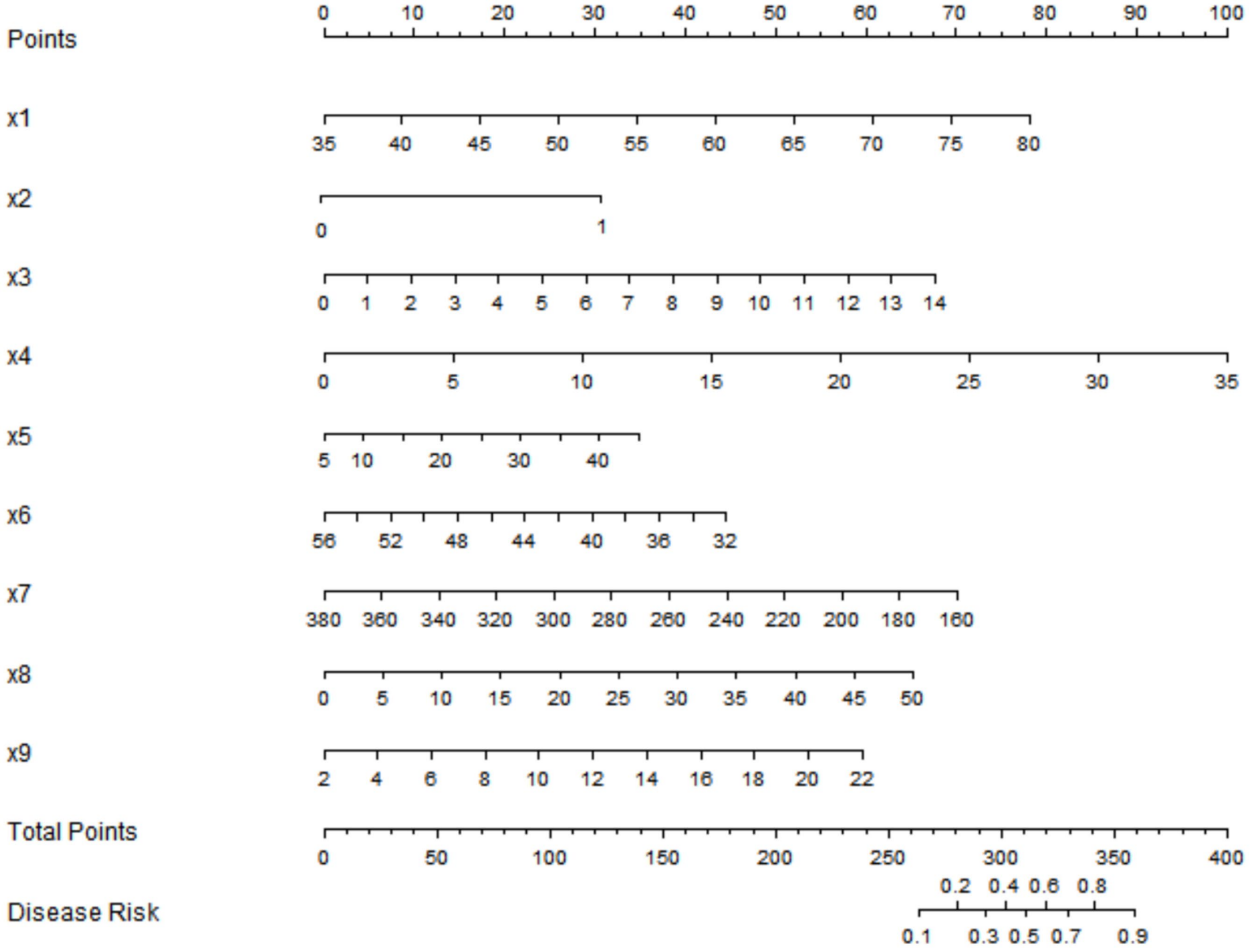
Nomogram prediction model for myocardial injury in advanced gastric cancer treated with fluorouracil combined with platinum-based drugs (x1: age, x2: hypertension, x3: CRP, x4: IL-6, x5: TNF-α, x6: serum albumin, x7: prealbumin, x8: Lp(a), x9: Hcy). Each predictor has a corresponding point scale. Locate the value of each predictor on the axis, draw a vertical line upward to the “Points” axis to get the points of the predictor, sum the points of all predictors to get the total score, and then draw a vertical line downward from the “Total Points” axis to the “Probability of Myocardial Injury” axis to obtain the probability of myocardial injury in the patient. Evaluation and validation of the nomogram prediction model for myocardial injury.

Based on the independent influencing factors identified through multivariate logistic regression analysis, a nomogram model was constructed to predict the risk of myocardial injury in patients with advanced gastric cancer treated with fluorouracil combined with platinum-based chemotherapy. Corresponding point scales were assigned to each factor according to their regression coefficients, with the total points corresponding to the probability of myocardial injury occurrence (Figure 1).

**Figure 2:**
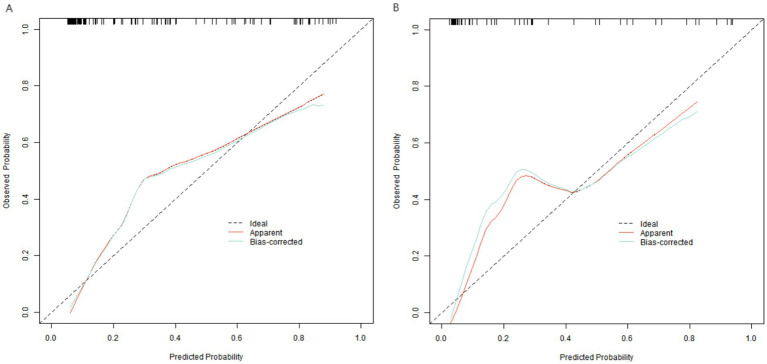
Calibration curves of nomogram prediction model (**A**: training set, **B**: validation set). The *x*-axis represents the predicted probability of myocardial injury, and the *y*-axis represents the actual probability of myocardial injury. The “Ideal” curve represents a perfect prediction where the predicted probability is completely consistent with the actual probability. The “Apparent” curve represents the calibration result of the original model. The “Bias-corrected” curve represents the calibration result after bootstrap validation.

**Figure 3:**
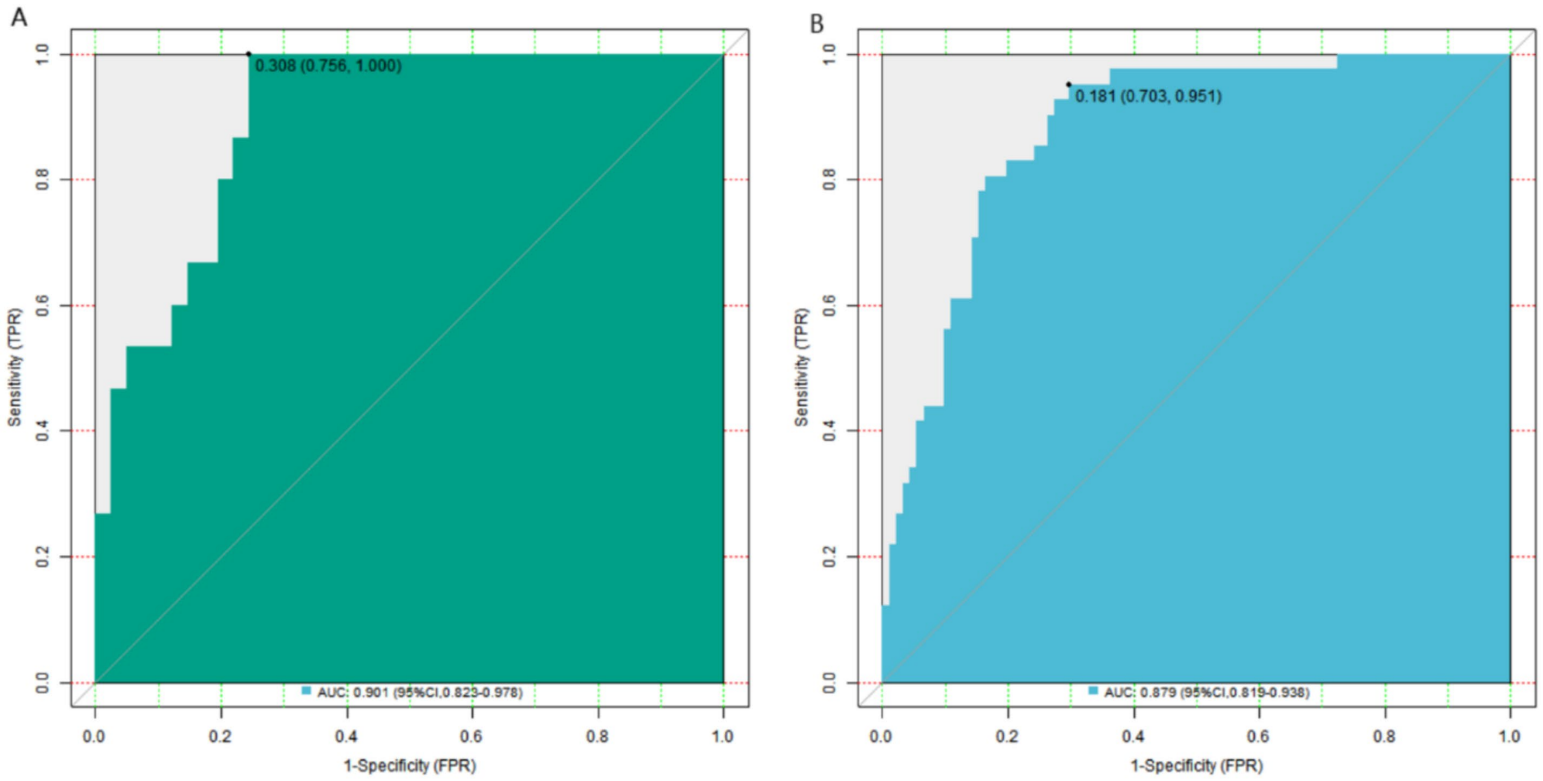
Receiver operating characteristic curves of nomogram prediction model (**A**: training set, **B**: validation set). The *x*-axis represents 1-specificity (false positive rate), and the *y*-axis represents sensitivity (true positive rate).

In the training set and validation set, the *C*-index indices of the constructed nomogram prediction model were 0.901 and 0.879, respectively. Further analysis through the calibration curve shows that the consistency between the model’s predicted values and the actual observed values is good, specifically manifested as the average absolute errors being 0.133 and 0.115, respectively. Furthermore, the results of the Hosmer–Lemeshow test indicated that the *χ*^2^ values of the training set and the validation set were 12.336 (*p* = 0.136) and 5.800 (*p* = 0.669), respectively (Figure 2). In addition, ROC curve analysis revealed the ability of the nomogram model to predict myocardial injury in patients with advanced gastric cancer who received fluorouracil combined with platinum-based drugs. The AUC of the training set and the validation set were 0.901 (95% CI: 0.823–0.978) and 0.879 (95% CI: 0.819–0.938). The corresponding sensitivity and specificity combinations were 0.756, 1.000 and 0.703, 0.951, respectively (Figure 3). 1,000 resamples were conducted in the bootstrap validation, and the *C*-index after validation was 0.865, which was close to the original *C*-index of 0.883, suggesting good model stability and no significant overfitting. Comparison of predictive performance showed that no single independent risk factor had predictive power comparable to the combined model. For example, the OR values of individual factors ranged from 1.059 (TNF-α) to 1.290 (CRP), while the combined model achieved an AUC of 0.879 in the training set and 0.901 in the validation set, confirming that integrating multiple factors significantly improves the accuracy of myocardial injury prediction.

### Decision curve analysis of the myocardial injury prediction nomogram model

**Figure 4:**
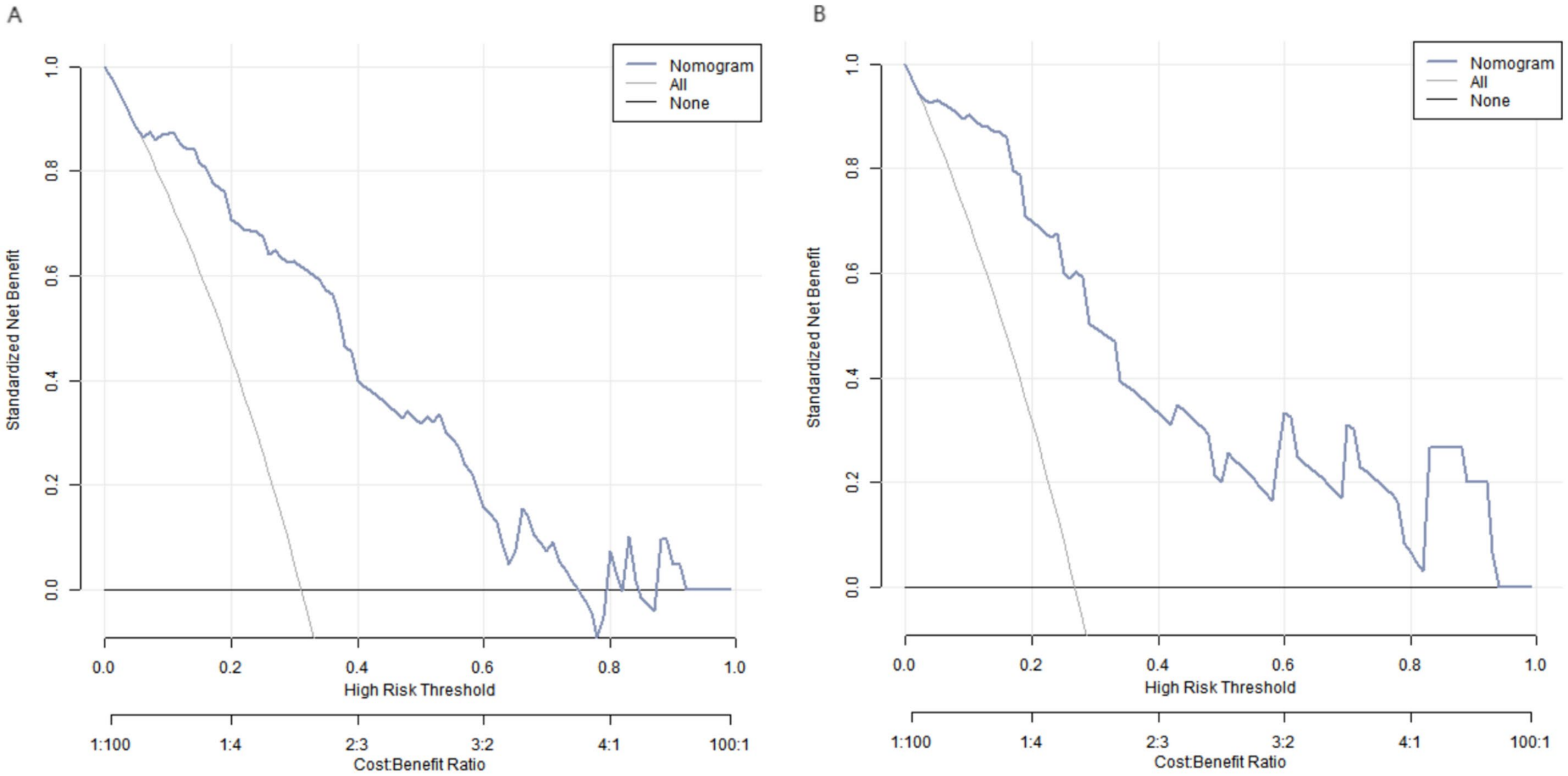
Decision curves of nomogram prediction model (**A**: training set, **B**: validation set). The *x*-axis represents the high-risk threshold probability of myocardial injury, and the *y*-axis represents the net benefit. The “Nomogram” curve represents the net benefit of using the nomogram model to predict myocardial injury. The “All” curve represents the net benefit of assuming all patients have myocardial injury, and the “None” curve represents the net benefit of assuming no patients have myocardial injury.

The DCA showed that when the threshold probability is approximately between 0.05 and 0.75, using the nomogram model constructed in this study to predict myocardial injury provides greater clinical benefit than the strategies of assuming either all patients have myocardial injury or none have myocardial injury preoperatively (see Figure 4).

## Discussion

Gastric cancer is a leading cause of cancer-related mortality globally (4). The chemotherapy regimen combining fluorouracil with platinum-based drugs is a commonly used treatment for advanced gastric cancer (5). Although this approach can prolong patient survival to some extent, the cardiotoxicity associated with the regimen cannot be overlooked, as it may lead to myocardial damage and negatively impact patients’ quality of life and prognosis (6). Currently, chemotherapy-related myocardial injury has received some attention, but the construction and evaluation of predictive models for myocardial injury in patients with advanced gastric cancer treated with fluorouracil combined with platinum-based drugs remain inadequate (7). This study aimed to construct a predictive model by analyzing baseline data and inflammation-nutrition-atherosclerosis factors, evaluate its clinical value, and provide a reference for clinical practice.

In this study, myocardial injury was observed in 56 cases (29.79%) in the training set and 23 cases (28.75%) in the validation set. There was no statistically significant difference in the incidence of myocardial injury or clinical characteristics between the training and validation sets (*p* > 0.05), indicating the rationality of the grouping. This ensures that the model constructed from the training set is well-representative and can be effectively validated in the validation set. The univariate and multivariate logistic regression analyses of the training set revealed that age, comorbid hypertension, CRP, IL-6, TNF-α, serum albumin, prealbumin, Lp(a), and Hcy were independent risk factors for myocardial injury in patients with advanced gastric cancer treated with fluorouracil combined with platinum-based chemotherapy. The association between age and myocardial injury may be attributed to the physiological degeneration of cardiac structure and function with aging, leading to reduced tolerance of cardiomyocytes to chemotherapeutic drugs (8). Hypertensive patients often have a heart under prolonged high load and impaired vascular endothelial function, making them more susceptible to myocardial injury when stimulated by chemotherapy drugs (9). The role of inflammatory factors CRP, IL-6, and TNF-α in myocardial injury is also crucial (10). CRP is an acute-phase reactive protein that significantly increases during inflammatory responses (11). Elevated levels of CRP not only indicate the presence of an inflammatory state in the body but also directly participate in the inflammatory response, damaging vascular endothelial cells and promoting the onset and progression of atherosclerosis, thereby increasing the risk of myocardial injury (12). IL-6 and TNF-α, as pro-inflammatory cytokines, can affect the function of cardiomyocytes through various pathways (13, 14). They can induce apoptosis of cardiomyocytes, suppress myocardial contractility, and also activate inflammatory cascades, leading to inflammatory infiltration and damage in myocardial tissue. During chemotherapy, the death of tumor cells can further stimulate the release of inflammatory factors, exacerbating the inflammatory response and increasing the likelihood of myocardial injury (15). Serum albumin and prealbumin are important indicators reflecting the body’s nutritional status (16, 17). Low levels of serum albumin and prealbumin indicate poor nutritional status in patients. Malnutrition can impair the energy metabolism and repair capacity of myocardial cells, reduce the heart’s tolerance to chemotherapeutic drugs, and thereby increase susceptibility to myocardial injury (18). Lp(a) and Hcy are factors closely associated with atherosclerosis (19). Lp(a) has atherogenic and prothrombotic properties. It can deposit in the vascular wall, promote inflammatory responses and plaque formation, impair coronary artery blood flow, and increase the risk of myocardial ischemia and injury (20). Hcy is a sulfur-containing amino acid, and elevated levels can lead to vascular endothelial cell damage, increased oxidative stress, and a heightened tendency for thrombosis, thereby increasing the risk of myocardial injury (21). Based on the aforementioned independent risk factors, this study constructed a nomogram prediction model. The nomogram integrated multiple risk factors in an intuitive graphical format, enabling clinicians to quickly assess the probability of myocardial injury in patients. The model demonstrated good calibration and fit in both the training and validation sets. The *C*-index values were 0.883 and 0.906, respectively, indicating high discriminative ability to distinguish between patients with and without myocardial injury. The mean absolute errors between predicted and observed values were 0.133 and 0.115, and the Hosmer–Lemeshow test yielded *p*-values of 0.136 and 0.669, further confirming the strong agreement between predicted and actual outcomes, as well as the model’s excellent fit. ROC curve analysis showed that the AUCs for predicting myocardial injury in the training and validation sets were 0.901 (95% CI: 0.823–0.978) and 0.879 (95% CI: 0.819–0.938) and respectively, with sensitivities and specificities of 0.756, 1.000 and 0.703, 0.951, respectively. An AUC closer to 1 indicates higher predictive accuracy, and the high AUC values in both datasets suggest that the model has strong predictive capability for myocardial injury. The high specificity means the model can accurately identify patients without myocardial injury, reducing unnecessary interventions, while the sensitivity—though slightly lower than specificity—remains within an acceptable range, detecting most cases of myocardial injury. The optimal threshold for the validation set was determined using the Youden index. When the threshold probability was 0.308, the Youden index reached the maximum value (0.756), so this value was selected as the optimal threshold. Clinically, the high specificity (1.000) can avoid unnecessary interventions for patients without myocardial injury, while the sensitivity (0.756) can detect most patients with myocardial injury. Combining the model with regular dynamic monitoring of cardiac function indicators (e.g., ECG, echocardiography, cTn) can further reduce the risk of missed diagnoses and optimize clinical decision-making. DCA revealed that when the threshold probability ranged between approximately 0.05 and 0.94, using the nomogram model for predicting myocardial injury provided greater clinical benefit than assuming all patients either had or did not have myocardial injury. This suggests that the model can offer valuable decision-making support in clinical practice, helping physicians tailor treatment plans more rationally based on individual patient risk, thereby avoiding overtreatment or undertreatment and ultimately improving therapeutic outcomes and prognosis.

However, this study had several limitations. Firstly, the number of myocardial injury events in the training set was insufficient. The final model includes 9 independent variables, and according to the EPV principle (requiring 10–20 events per variable), 90–180 myocardial injury events are needed in the training set. However, only 56 events were observed (≈6.2 events per variable), which may affect the model’s robustness. Although bootstrap validation showed certain stability, future multicenter studies with larger sample sizes are needed to increase the number of myocardial injury events and meet the EPV requirement, thereby improving the model’s reliability. Secondly, lack of an independent test set and external validation restricts the model’s generalizability to broader populations. Additionally, the study only included patients from a single center over a specific period, which may limit the representativeness of the sample. Variations in genetic background, lifestyle, and disease characteristics among patients from different regions and ethnicities could influence the risk of myocardial injury and the model’s predictive accuracy. In subsequent research, we will conduct a multicenter study, collect more samples to establish an independent test set, and perform external validation to further confirm the model’s performance and avoid overfitting. Thirdly, the lack of systematic recording of oxaliplatin dose delays/reductions due to non-cardiac toxicities. Although a post-hoc analysis using limited data showed no significant difference in myocardial injury incidence between patients with potential dose adjustments and those without, the missing detailed dose adjustment information prevents us from fully excluding its potential confounding effect on the model. Future studies should optimize data collection to record all chemotherapy dose changes, to more rigorously validate the model’s stability. Fourthly, the lack of formal verification of non-linear relationships between continuous predictors and myocardial injury. Although clinical evidence and model performance indirectly support the rationality of continuous variable processing, we plan to use restricted cubic spline analysis to systematically verify linearity in subsequent multicenter studies, to further optimize the model’s statistical basis.

Despite these limitations, the prediction model for myocardial injury in patients with advanced gastric cancer treated with fluorouracil and platinum-based chemotherapy—constructed based on baseline data and inflammation-nutrition-atherosclerosis factors—holds significant clinical value. It facilitates early risk prediction, providing clinicians with a reference for personalized treatment strategies to minimize myocardial injury risk and improve patient outcomes. In future clinical practice and research, further validation and refinement of this model are warranted to enhance its utility in supporting the treatment and management of gastric cancer patients.

## Conclusion

This predictive model aids in the early identification of myocardial injury, guiding clinical decision-making and improving prognosis.

## Data Availability

The original contributions presented in the study are included in the article/supplementary material, further inquiries can be directed to the corresponding author.
